# Appointment

**Published:** 1867-03

**Authors:** 


*Appointment.*—WILLIAM LEWITT, M.D., has been appointed Demonstrator of Anatomy in Rush Medical College, in the place of R. M. Lackey, M.D., resigned. Dr. Lewitt is well known to a large part of the profession and medical students of the North-west, having for many years past filled the same position in the University of Michigan. His qualifications for the post have been demonstrated before the great classes of that institution—his executive ability being sufficiently shown in the fact that he has annually supplied and directed the labors of over one hundred dissecting classes. The dissecting rooms under his control have been models of neatness and order —it is safe to say, being in all respects, thus far, unsurpassed on the continent. No more beautiful anatomical preparations can be found than those he has personally made. An accurate and minute anatomist, a rapid and expert dissector, courteous and attentive to his classes, the College is to be congratulated upon its success in securing his services. Having enjoyed the respect and confidence of a large circle of private patients in Ann Arbor, and its vicinity, he will bring to this new sphere of effort large guarantees of successful practice, both as a physician and surgeon.

It is expected that Dr. Lewitt will remove to this city about the first of May, ensuing. Personally, and as connected with the College, the Editor acknowledges unmeasured delight at Dr. Lewitt's acceptance of the position. Meanwhile, he extends his sincerest sympathies to the few remaining members of the Medical Department of the University of Michigan.

